# High Interferon Signature Leads to Increased STAT1/3/5 Phosphorylation in PBMCs From SLE Patients by Single Cell Mass Cytometry

**DOI:** 10.3389/fimmu.2022.833636

**Published:** 2022-01-28

**Authors:** Gloria Yiu, Tue Kruse Rasmussen, Brandon L. Tsai, Vivian K. Diep, David J. Haddon, Jennifer Tsoi, Gopika D. Miller, Begoña Comin-Anduix, Bent Deleuran, Gay M. Crooks, Paul J. Utz

**Affiliations:** ^1^ Department of Medicine, Division of Immunology and Rheumatology, Stanford School of Medicine, Stanford, CA, United States; ^2^ Department of Rheumatology, University of California, Los Angeles, Los Angeles, CA, United States; ^3^ Department of Biomedicine, Aarhus University, Aarhus, Denmark; ^4^ Department of Rheumatology, Aarhus University Hospital, Aarhus, Denmark; ^5^ Department of Human Genetics, University of California, Los Angeles, Los Angeles, CA, United States; ^6^ Department of Surgery David Geffen School of Medicine, Johnson Comprehensive Cancer Center, University of California, Los Angeles, Los Angeles, CA, United States; ^7^ Jonsson Comprehensive Cancer Center, University of California, Los Angeles (UCLA), Los Angeles, CA, United States; ^8^ Parker Institute for Cancer Immunotherapy, San Francisco, CA, United States; ^9^ Department of Pathology & Laboratory Medicine, David Geffen School of Medicine, University of California, Los Angeles, Los Angeles, CA, United States; ^10^ Eli and Edythe Center of Regenerative Medicine and Stem Cell Research, University of California, Los Angeles (UCLA), Los Angeles, CA, United States; ^11^ Division of Pediatric Hematology-Oncology, Department of Pediatrics, David Geffen School of Medicine, University of California, Los Angeles (UCLA), Los Angeles, CA, United States; ^12^ Institute for Immunity, Transplantation, and Infection, Stanford University School of Medicine, Stanford, CA, United States

**Keywords:** interferon, lupus (SLE), CyTOF mass cytometry, STAT3 (signal transducer and activator of transcription 3), STAT1, autoantibodies, interferon signature

## Abstract

The establishment of an “interferon (IFN) signature” to subset SLE patients on disease severity has led to therapeutics targeting IFNα. Here, we investigate IFN signaling in SLE using multiplexed protein arrays and single cell cytometry by time of flight (CyTOF). First, the IFN signature for SLE patients (n=81) from the Stanford Lupus Registry is determined using fluidigm qPCR measuring 44 previously determined IFN-inducible transcripts. IFN-high (IFN-H) patients have increased SLE criteria and renal/CNS/immunologic involvement, and increased autoantibody reactivity against spliceosome-associated antigens. CyTOF analysis is performed on non-stimulated and stimulated (IFNα, IFNγ, IL-21) PBMCs from SLE patients (n=25) and HCs (n=9) in a panel identifying changes in phosphorylation of intracellular signaling proteins (pTOF). Another panel is utilized to detect changes in intracellular cytokine (ICTOF) production in non-stimulated and stimulated (PMA/ionomycin) PBMCs from SLE patients (n=31) and HCs (n=17). Bioinformatic analysis by MetaCyto and OMIQ reveal phenotypic changes in immune cell subsets between IFN-H and IFN-low (IFN-L) patients. Most notably, IFN-H patients exhibit increased STAT1/3/5 phosphorylation downstream of cytokine stimulation and increased phosphorylation of non-canonical STAT proteins. These results suggest that IFN signaling in SLE modulates STAT phosphorylation, potentially uncovering possible targets for future therapeutic approaches.

## Introduction

Systemic Lupus Erythematosus (SLE) is a chronic autoimmune disease that carries significant clinical burden for patients including organ damage and death (1–3). The disease is clinically characterized by periods of remission or flare involving inflammation in multiple organs, including skin, kidney, vasculature, and others. A hallmark of SLE is the production of antibodies against self-associated antigens, or autoantibodies. Elucidation of SLE pathogenesis and development of effective therapies is limited by both the clinical and biological heterogeneity seen in patients (4).

Multiple studies support a role for type I interferons, and IFNα, specifically, in SLE. Transcript profiling of peripheral blood mononuclear cells (PBMCs) (5) and immune cell subsets (6) from SLE patients demonstrate the presence of an IFNα-inducible gene expression signature in a subset of patients that correlates with clinical SLE criteria and autoantibody production (7). Recent single cell RNA-seq analysis of kidney biopsies from lupus nephritis (LN) patients reveals an IFN response signature in infiltrating immune cells that correlates with the same signature in the peripheral blood (8, 9). These studies establish the IFN signature as pathogenic in SLE and support its use to subset patients as IFN-high (IFN-H) or IFN-low (IFN-L). Because IFN-H patients demonstrate more severe disease manifestations, IFNα has become a therapeutic target in SLE. Consequently, fully humanized monoclonal antibodies against subunit 1 of the type I interferon receptor (IFNAR1), and its administration is associated with significantly reduced overall disease activity (BICLA) and corticosteroid use, and improved dermatitis (10). In August 2021, the Food and Drug Administration approved this class of treatment for moderate to severe SLE, marking only the third approved for SLE in the last 50 years (11, 12). How IFN signaling interacts with other immune pathways in SLE remains unclear.

In addition to IFNα, numerous other cytokines are dysregulated in SLE. Elevated levels of Type II IFN, IFNγ, have been reported in both murine and human SLE and is associated with more severe disease (13–15). Murine models of SLE, genetically modified to overexpress IFNγ (16) or receiving endogenous IFNγ (17) exhibit more severe organ specific disease that is reversible with IFNγ targeting. Urine proteomic profiling of LN patients have also demonstrated that patients with more severe kidney disease display chemokine profiles induced by IFNγ (18). Notably, type I IFNs like IFNα can activate natural killer (NK) cells cytotoxicity and IFNγ production, and 25% of IFNγ-inducible genes overlap with IFNα-inducible genes (19). Recent work has established the role of follicular helper T cells (Tfhs) in autoimmune diseases including SLE due to Tfh function in germinal centers (GC) (20) – a key site for antibody maturation and autoantibody production (21). Tfh cells produce high levels of IL-21, which is necessary for GC formation and Tfh differentiation (22). PBMCs isolated from SLE patients express higher levels of IL-21 compared to HCs (23), and peripheral follicular T helper cells are reportedly expanded in SLE patients and induce B cell differentiation into plasmablasts *via* IL-21 (24).

Many cytokines dysregulated in SLE including IFNα, IFNγ, and IL-21 signal *via* shared pathways involving the phosphorylation of Signal Transduction and Activators of Transcription (STAT) family members. Downstream of receptor ligation, Janus family tyrosine kinases (JAKs) proteins phosphorylate STATs leading to translocation to the nucleus and induction of gene programs crucial to immune function. STAT phosphorylation downstream of IFNα, IFNγ, or IL-21 receptor engagement is well described, where IFNα most frequently signals *via* pSTAT1/pSTAT2 (and to lesser degrees pSTAT3, pSTAT4, pSTAT5), IFNγ signals *via* a pSTAT1 homodimer, and IL-21 signals most frequently *via* pSTAT1/pSTAT3 (and to lesser degrees pSTAT5) (25–27). Phospho-specific flow cytometry is a well-established technique that can simultaneously quantify activation of multiple STAT proteins, in response to multiple cytokines and in various cell types. However, its flow cytometry foundations and consequent restrictions of fluorescence-based spectral overlap limit the multiplexing ability of phosphoflow. Technological advances have allowed scientists to design highly multiplexed panels to simultaneously measure more than 40 markers by using single cell mass cytometry by time-of-flight (CyTOF) (28). CyTOF is a hybrid mass spectrometer-flow cytometer that employs transition metal isotope reporters – not found in biological samples. This approach permits for the unprecedented single-cell analysis of surface markers and intracellularly functional proteins including STAT proteins. CyTOF’s capacity for high-resolution immunophenotyping makes it the ideal tool for understanding a complex disease like SLE, characterized by global dysregulation of numerous immune cell subsets, and signaling pathways.

Here we present a single-cell proteomic study of IFNα, IFNγ, and IL-21 signaling pathways in SLE patients stratified by the IFN signature. We find that IFN-H patients have more severe disease and increased levels of autoantibodies against spliceosome associated antigens compared to IFN-L patients. CyTOF analysis using established bioinformatic approaches identifies single cell differences in cellular phenotype, cytokine signaling and immune function between IFN-H and IFN-L patients. Utilizing this technology, we report increased phosphorylation of both common and less-common STAT proteins associated with IFN signature in SLE. This work sheds light on interactions between IFN signaling and other immunologically crucial pathways that signal *via* STAT proteins. These insights could hold implications on how IFNα inhibition could modulate other signaling pathways, thereby uncovering possible targets for future therapies.

## Results

### Interferon Profiling of SLE Patients and Healthy Controls Reveals IFN Signature in SLE Patients

**Figure 1:**
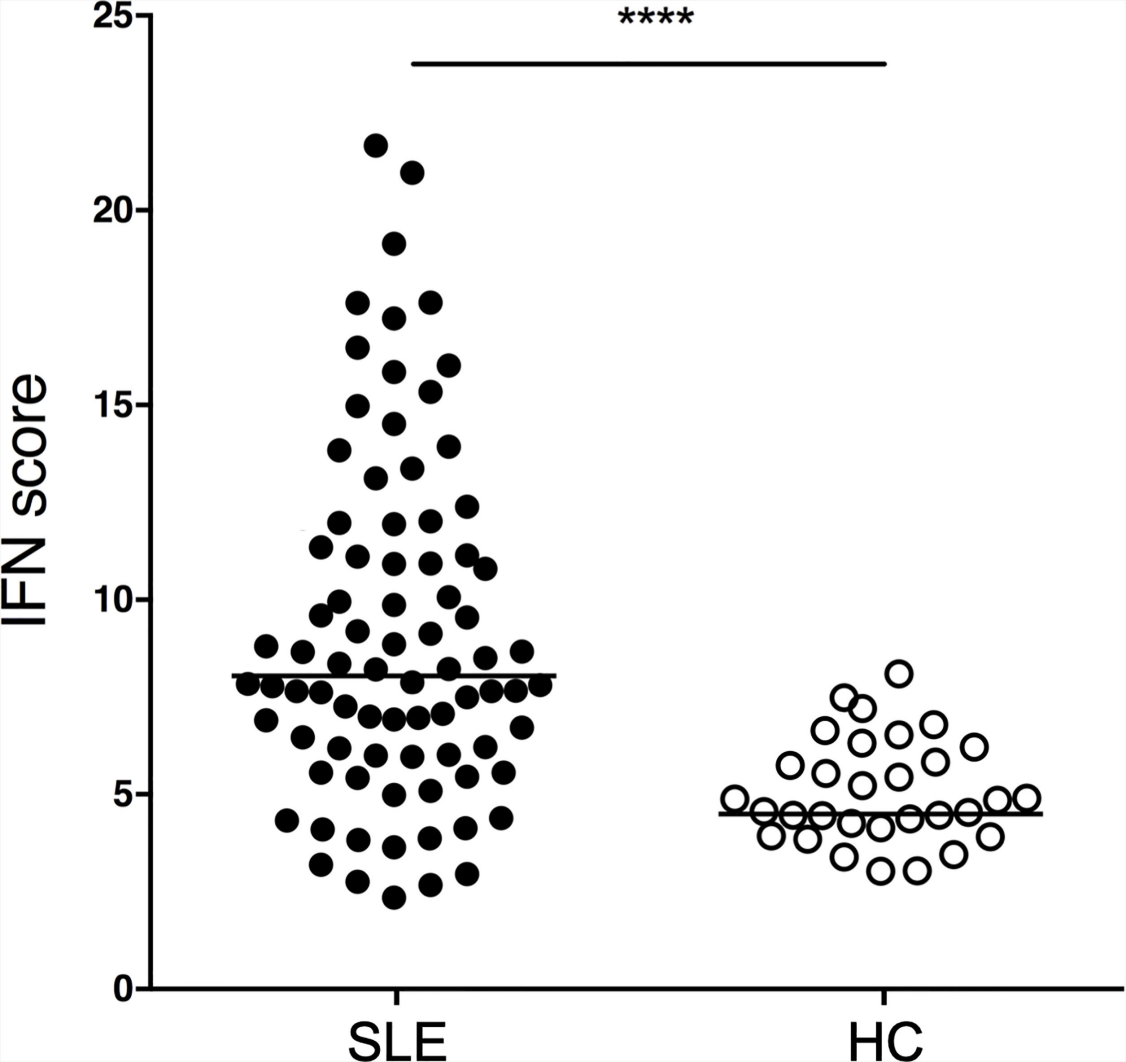
IFN scores of SLE patients and HCs. SLE patients (n=81) and HCs (n=31) were profiled for an IFN signature using the transcripts shown in **Supplementary Table 1**. p<0.0001. P value was determined by Mann-Whitney test. SLE patients had a median IFN biosignature of 8.0 (IQR 5.9-11.5) while HCs had a median of 4.5 (IQR 4.0-5.5).

Sourcing across five established studies (5, 6, 29–31), we have designed a consensus panel of 44 IFN-inducible transcripts (**Supplementary Table 1**) for profiling RNA from patients (n=81) in the Stanford SLE Registry (**Table 1**) and HCs (n=26). Each gene is normalized to the maximum value across all samples. A combined score is calculated by adding the values of all genes for each sample (**Figure 1**).

**Table 1:** Demographic and Patient characteristics of the patients*.

	IFN signature (n=81)	CyTOF (n=31)
Age (years)	42.0 +/- 11.2	41.8 +/- 10.1
Female sex – no. (%)	75 (92)	29 (94)
Race** – no. (%)		
White	28 (34)	5 (16)
Black	2 (2.5)	1 (3.2)
Asian	22 (27)	12 (39)
Latino	19 (23)	11 (35)
Other	12 (15)	5 (16)
SLE criteria – no. (%)		
All	4.49+/- 4.04	5.1+/- 3.4
Malar Rash	32 (40)	15 (48)
Discoid Rash	16 (20)	11 (35)
Photosensitivity	42 (52)	16 (52)
Oral/nasal ulcers	49 (60)	19 (61)
Arthritis	64 (79)	25 (80)
Pleurisy/pericarditis	18 (22)	8 (26)
Renal	43 (53)	12 (39)
Neurologic	13 (16)	7 (23)
Hematologic	28 (35)	11 (35)
Immunologic	45 (56)	14 (45)
+ ANA	72 (89)	26 (84)
History of low C3/C4	51 (63)	22 (71)
History of +anti-dsDNA	32 (40)	12 (39)
History of +anti-Ro/SSA	14 (17)	8 (26)
History of +anti-La/SSB	7 (8.6)	2 (6.4)
History of +anti-Smith	22 (27)	6 (20)
History of +anti-RNP	14 (17)	6 (20)
Medications – no. (%)		
Glucocorticoid	48 (59)	16 (52)
Plaquenil	66 (81)	24 (77)
DMARD***	14 (17)	5 (16)
Cellcept	18 (22)	5 (16)
Biologic****	8 (9.9)	3 (9.7)

*Plus-minus values are means +/- SD. Percentages may not total 100 because of rounding.

**Race and ethnic groups were reported by patients.

***DMARD denotes disease modifying antirheumatic drug which includes azathioprine, methotrexate, sulfasalazine, and leflunomide.

****biologic denotes monoclonal antibody treatments including belinumab and rituximab.

SLE patients exhibit significantly higher IFN scores as compared to HCs. (p<0.0001, Mann-Whitney). SLE patients have a median IFN signature of 8.0 (IQR 5.9-11.5) while HCs have a median of 4.5 (IQR 4.0-5.5). The IFN signature is calculated using our consensus panel and shows excellent correlation to two other published interferon signature scores (rho=0.98, p<0.0001 and rho=0.92, p<0.0001, respectively) (**Supplementary Figure 1**). Using our consensus IFN signature, SLE patients are subsetted as IFN-H (upper quartile) and IFN-L (lower quartile).

### The Interferon Signature Is Associated With Clinical Manifestations

To determine the relevance of interferon signature to clinical manifestations in the Stanford SLE cohort, a retrospective chart review is performed on unique patients (n=42) by a senior rheumatology fellow (GDM) who is blinded to the IFN signature status of each patient. The review extracts 52 clinical parameters and medication regimens for patients for up to 5 years of clinical care.

**Figure 2:**
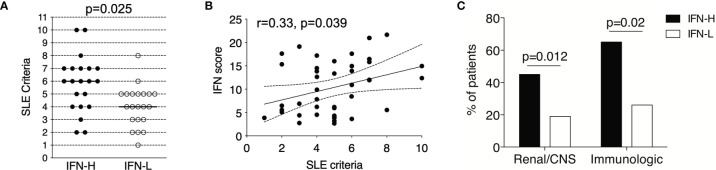
**(A-C)** IFN signature in SLE patients is associated with more severe clinical manifestations. **(A)** IFN-H (n=21) patients exhibited significantly increased SLE criteria as compared to IFN- L (n=21) patients (p=0.025), where IFN-H patients had a median of 6 SLE criteria and IFN-L patients had a median of 4 SLE criteria. Criteria used followed the 1982 American College of Rheumatology SLE revision. P values determined by Mann-Whitney test. **(B)** IFN score and SLE criteria are significantly positively associated by Spearman regression analysis (r=0.33, p=0.049). 95% confidence interval is indicated by dashed lines. **(C)** IFN-H patients exhibit significantly higher percentage of renal (proteinuria and/or biopsy-proven LN) or central nervous system (CNS=central nervous system as defined as seizure, psychosis, and/or cerebritis), p=0.012; and immunologic (immunologic as defined as dsDNA+ or Sm+) p=0.02, manifestations as compared to IFN-L patients. P values determined by Chi-Square test.

IFN-H as compared to IFN-L patients exhibit significantly increased SLE disease manifestations as characterized by the SLE criteria (p=0.01) at the time of PBMC collection. IFN-H patients have a median of six SLE criteria and IFN-L patients have a median of four SLE criteria (**Figure 2A**). Increasing SLE score is also significantly and positively associated with SLE criteria, p=0.049 (**Figure 2B**). Furthermore, IFN-H patients have significantly increased frequency of organ and/or hematologic disease. These disease manifestations include increased frequency of renal, neurological or immunologic involvement (**Figure 2C**). Renal disease is defined as 24 hr urine protein representing >= 500 mg of protein/24-hour, red blood cell casts, or biopsy proven LN. Neurological disease is defined as psychosis, seizure or cerebritis. Immunologic involvement is defined as dsDNA+ or Sm+.

### IFN-H Patients Have Increased Levels of Autoantibodies Against SLE-Associated Antigens

We employ protein microarrays to profile autoantibodies in HCs (n=20) and SLE patients (n=73) stratified by IFN signature. This method has been well-established by our lab for studying human (32–34) and murine SLE (35–37). The arrays contained 24 unique features that are known or putative autoantigens in SLE. Significance Analysis of Microarrays (SAM) algorithm determines antigens with statistically significant differences between groups of mice and a hierarchical clustering program groups individual subjects based on similar autoantibody profiles (38).

**Figure 3:**
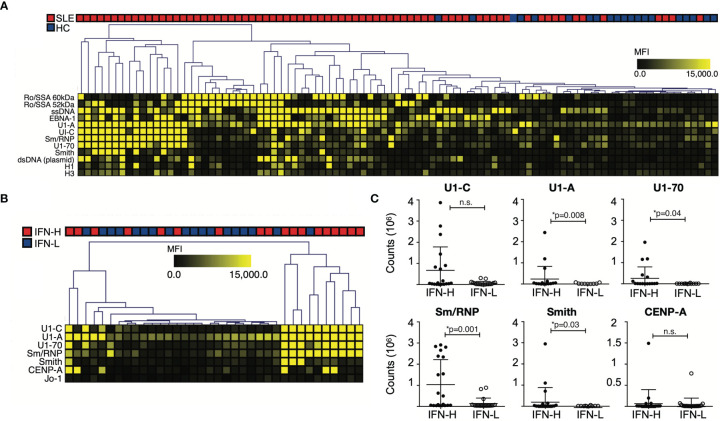
**(A-C)** IFN signature associated with increased autoantibody reactivity against known SLE-associated associated antigens. **(A)** Autoantibody profiling of SLE patients versus HCs. SLE patients exhibit increased autoantibody production against SLE-associated antigens. **(B)** SLE patients are stratified based on IFN signature. IFN-H patients exhibit increased autoantibody reactivity against spliceosome-associated antigens. **(A, B)** Individual autoantigen arrays were incubated with serum obtained from SLE patients or HCs. Color intensity of each grid in heatmap reflects mean fluorescence intensity (MFI). The SAM algorithm was used to determine antigen features with statistically significant differences in reactivity between sera derived from two groups. Hierarchical clustering of samples based on reactivity to antigens with statistically significant differences is displayed as a heatmap and dendrogram. **(C)** ELISA confirmation of SAM identified autoantibodies against antigens with statistically significant differences between IFN-H and IFN-L groups. Counts are calculated by subtracting BSA from average. U1-A, p=0.008; U1-70, p=0.04; Sm/RNP, p=0.001; Smith, p=0.03. IFN-H patients have increased autoantibody production against U1-C, though not statistically significant p=0.55. P values were determined by Mann-Whitney test. ns, not significant.

As expected, IgM and IgG autoantibody reactivity against SLE-associated autoantigens is increased in SLE patients as compared to HCs (**Figure 3A**). SAM identified 12 of these autoantibodies as significantly increased in SLE patients as compared to HCs. These include Ro/SSA, ssDNA, EBNA-1, spliceosome components U1-A, U1-C, U1-70 and Sm/RNP, Smith, dsDNA (plasmid) and histones.

When comparing IFN-H to IFN-L patients, IFN-H patients exhibit increased autoantibody production predominantly against spliceosome components: U1-C, U1-A, U1-70, Sm/RNP in addition to, Smith, CENP-A and Jo-1 (**Figure 3B**). These significant array findings are validated using enzyme-linked immunosorbent assay (ELISA) (**Figure 3C**). Jo-1 is not validated because of low reactivity across both groups. Of the remaining six antigens, four are identified by ELISA to have significant differences between IFN-H (n=20) and IFN-L (n=20) patients (U1-A, p=0.008; U1-70, p=0.04; Sm/RNP, p=0.001; Smith, p=0.03). IFN-H patients have increased autoantibody production against U1-C, though not significant (p=0.55). Data for HC (n=3) not shown.

### Mass Cytometry Identifies Differences in Abundance of Immune Subsets in IFN-H Versus IFN-L PBMCs

To understand global differences in immune cells and signaling between IFN-H and IFN-L patients, we have utilized two mass cytometry panels – one panel designed to detect changes in phosphorylation of intracellular signaling proteins (pTOF) and another panel to detect changes in intracellular cytokine (ICTOF) production (see **Supplementary Table 2** for specific markers and panel design). For pTOF analysis, PBMCs from IFN-H (n=13), IFN-L (n=12) and HC (n=9) patients are stimulated with IFNα, IFNγ, IL-21 or unstimulated for 15 minutes then analyzed by mass cytometry. For ICTOF, PBMCs from IFN-H (n=15), IFN-L (n=16), and HC (n=17) patients are stimulated with phorbol myristate acetate (PMA) and ionomycin (ION) for 4 hours then analyzed by mass cytometry.

**Figure 4:**
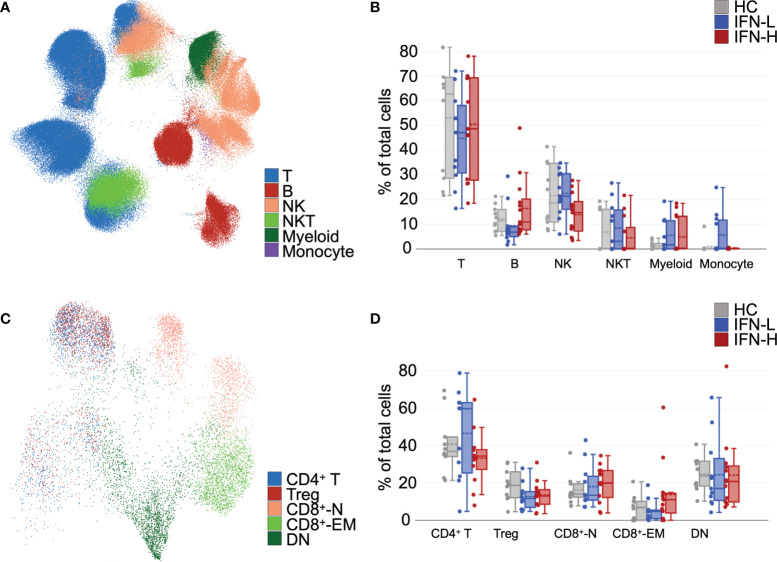
**(A-D)** Unsupervised analysis of mass cytometry identifies differences in abundances of immune cell and T cell subsets in SLE patients subsetted on high or low IFN signature. **(A)** Unsupervised uniform manifold approximation and projection (UMAP) and FlowSOM algorithms were utilized in OMIQ, which yielded distinct clusters in pTOF panel identified T cells, B cells, NK cells, NKT cells, myeloid cells, and monocytes. **(B)** Abundances of immune cell subsets identified in **(A)** from unstimulated samples. **(C)** UMAP analysis of T cells identifies multiple T cell subsets (CD4^+^ T cells, Tregs, CD8^+^-N = naïve CD8^+^ T cells, CD8^+^-EM = effector memory CD8^+^ T cells, and DN = double negative T cells). **(D)** Abundances of T cell subsets identified in **(C)** from unstimulated samples. Tregs were manually gated in OMIQ on CD25^+^CD127^-^.

To evaluate immune cell subsets, unsupervised uniform manifold approximation and projection (UMAP) (39) and FlowSOM (40) algorithms are utilized in OMIQ (41). Our cluster analysis identified major immune cell subsets in both pTOF (**Figure 4A**) and ICTOF (**Supplementary Figure 2B**) panels. Clusters for pTOF panel identify T cells, B cells, NK cells, NKT cells, myeloid cells, and monocytes. As expected, cell subsets cluster analysis remains unchanged after 15-minute PBMCs stimulation with IFNα, IFNγ, or IL-21 (**Supplementary Figure 2A**). The abundance of B cells in IFN-H patients is higher as compared to IFN-L patients and HCs (**Figure 4B**). This finding is also replicated in analysis of the ICTOF panel (**Supplementary Figure 2D**). IFN-H patients also demonstrates lower NK cell abundance as compared to IFN-L and HCs. This result is not prominently seen in the ICTOF panel analysis. Differences in major cell populations between the two panels can be attributed to dissimilar surface markers utilized in pTOF and ICTOF panels that are necessary to accommodate functional markers available on different metals. Because our panels include more surface markers to distinguish T cell subsets, additional analysis is performed on only T cells for both pTOF (**Figure 4C**) and ICTOF (**Supplementary Figure 2C**) panels. Due to rarity of T regulatory (Tregs: CD25^+^CD127^-^), T follicular helper (Tfh: CXCR5^+^PD1^+^), and T helper 17 (CD45RA^-^IL17^+^) cell subsets, these subsets are manually gated in OMIQ. Analysis of pTOF T cells reveal a decreased abundance of Tregs in SLE patients as compared to HCs (**Figure 4D**), with no notable difference between IFN-H and IFN-L patients. This finding is also demonstrated in analysis of the ICTOF panel, which also reveals decreased abundance of Tfh cells in IFN-H patients as compared to IFN-L patients and HCs (**Supplementary Figure 2E**).

### Effect Size of IFN Signature on STAT1/3/5 Phosphorylation in Immune Cells

**Figure 5:**
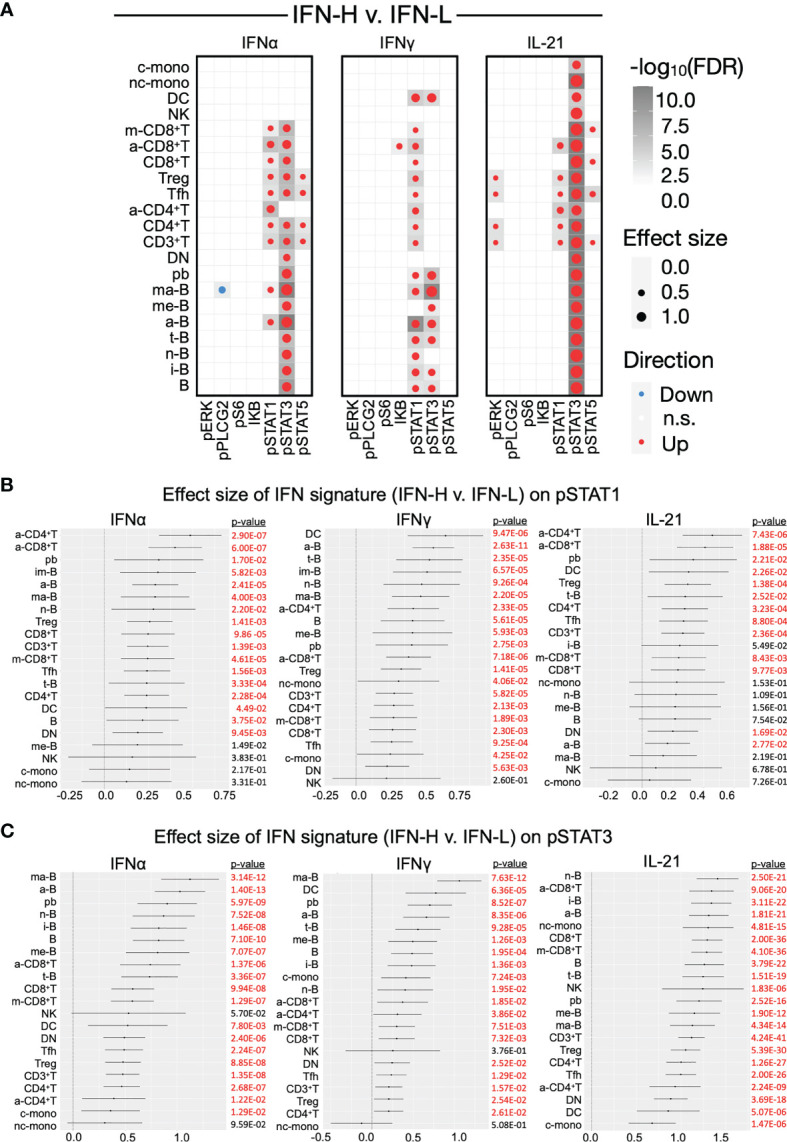
**(A-C)** Effect sizes of IFN signature on phosphorylated signaling in SLE PBMCs. **(A)** Dotmap represents effect size of IFN signature (IFN-H v. IFN-L patients) on signaling protein (columns) across cell subsets (rows) after stimulation with IFNα (left), IFNγ (middle), or IL-21 (right). Dot size depicts effect size, larger dots have greater effect sizes. Color depicts direction of effect size, red indicates positive effect size and blue indicates negative effect size. Shading of each box depicts statistical significance by -log10(FDR), darker boxes indicate greater statistical significance. Only cell populations with FDR < 0.05 are displayed in the dotmaps. Cell subsets (rows) are pre-defined by marker intensity **(** **Supplementary Table 3** **)**. **(B, C)** Forest plots representing the effect size of IFN signature (IFN-H v. IFN-L) on phosphorylation of pSTAT1 **(B)** and pSTAT3 **(C)** in cell subsets downstream of IFNα (left), IFNγ (middle), or IL-21 (right) stimulation. See **Supplementary Figure 3D** for forest plots for pSTAT5. Significant (p<0.05) effect sizes in red and not significant effect sizes in black. P values were adjusted using Benjamini-Hochberg false discovery rate (FDR).

To identify statistically significant differences between groups in our pTOF and ICTOF analysis, we independently analyze both panels with MetaCyto (42), an automated meta-analysis pipeline of cytometry datasets. MetaCyto accurately identifies cell populations across studies and applies hierarchical models to determine the effects of factors of interest on cell populations. This analysis requires “supervision,” where cell populations are pre-defined by panel markers (**Supplementary Table 3**). MetaCyto analysis of our pTOF panel demonstrates significant positive effect size of IFN signature (IFN-H v. IFN-L) on STAT1/3/5 phosphorylation across multiple cell subsets when comparing IFN-H to IFN-L patient samples (**Figures 5A–C** and **Supplementary Figure 3D**). Particularly notable are the pronounced positive effect sizes of IFN signature on phosphorylation of expected STAT proteins downstream of individual cytokine stimulations: IFNα/pSTAT1, IFNγ/pSTAT1, and IL-21/pSTAT3. Our results also demonstrate significant effect sizes of IFN signature (IFN-H v. IFN-L) on STAT proteins that are less frequently phosphorylated downstream of individual cytokine signaling: IFNα/pSTAT3, IFNα/pSTAT5, IFNγ/pSTAT3, and IL-21/pSTAT5. Together, these results suggest that the presence of a high IFN signature significantly effects the phosphorylation of frequently and less frequently used STAT proteins downstream of IFNα, IFNγ, and IL-21 signaling. Effect sizes of IFN signature (IFN-H v. IFN-L) on surface markers used in the pTOF analysis, and the effect size of SLE on expression of both surface and signaling markers are shown in **Supplementary Figure 3A-B**, respectively.

### Effect Size of IFN Signature on Intracellular Cytokine Production in Immune Cells

MetaCyto analysis of our ICTOF panel demonstrates a significant positive effect size of IFN signature (IFN-H v. IFN-L) on TNF-α and IFNγ production across all immune subsets, GM-CSF and perforin in a subset of B and T cells, IL-2 in T cells, IL17 in Tregs, Tfhs, and central memory CD4+ T cells, and IL-22 in multiple T cell subsets. Our analysis also reveals significantly negative effect size of IFN signature on IL-4 production in T-cells and IL-21 in both T and B cell subsets (**Supplementary Figure 3C**, right).

### IFN-H T Cells With Increased STAT1/3 Phosphorylation Express Stress and Co-Stimulation Proteins

**Figure 6:**
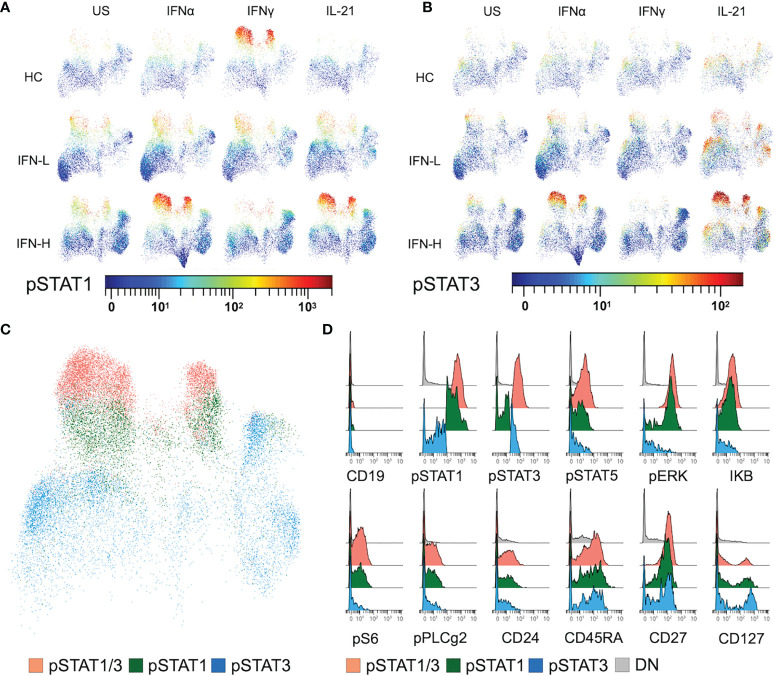
**(A-D)** IFN signature associated with increased STAT1/3 phosphorylation in T cells expressing stress, proliferation, and co-stimulation proteins. **(A, B)** UMAP plots of T cells isolated from HC, IFN-L and IFN-H patients stimulated with IFNα, IFNγ, IL-21 or unstimulated (US) colored by pSTAT1 **(A)** or pSTAT3 **(B)** intensity. **(C)** UMAP plot of T cells expressing pSTAT1 and 3 (orange), pSTAT1 (green), or pSTAT3 (blue). Plot of T cells from IFN-H samples stimulated with IFNα is representative of T cells from HC and IFN-L samples in all pTOF conditions. **(D)** Histograms displaying expression of both surface (CD19, CD24, CD27, and CD127) and intracellular signaling (pSTAT1, pSTAT3, pSTAT5, pERK, IKB, pS6, and pPLCg2) proteins of manually gated T cells expressing pSTAT1 and 3, pSTAT1, pSTAT3, or DN = double negative T cells, from IFN-H PBMCs stimulated with IFNα.

UMAP display of OMIQ analysis demonstrates an increased intensity of pSTAT1 downstream of IFNγ stimulation in HCs, which is absent in SLE patients regardless of IFN signature. T cells isolated from IFN-H patients show marked pSTAT1 intensity downstream of IFNα and IL-21 (**Figure 6A**) as compared to T cells isolated from IFN-L patients and HCs. These findings suggest a more prominent role of IFNα and IL-21 signaling in T cells as compared to IFNγ in SLE, and increased IFNα and IL-21 signaling associated with IFN signature. Similar analysis of pSTAT3 shows expected increased intensity of pSTAT3 downstream of IL-21 (as opposed to downstream of US, IFNα or IFNγ) in T cells isolated from HCs, IFN-L and IFN-H patients; with the most notable intensity in IFN-H T cells. While IFNα does not primarily signal through pSTAT3, stimulation of IFN-H T cells with IFNα displays increased STAT3 phosphorylation in IFN-H patients (**Figure 6B**).

Utilizing manual-gating of bi-axial plots in OMIQ, cells expressing high intensity of pSTAT1 (green), pSTAT3 (blue), or pSTAT1 and pSTAT3 (orange) are identified (**Figure 6C**) spanning multiple T cell subsets (**Figure 4C**). To further characterize T cells with high levels of STAT phosphorylation in response to IFNα, IFNγ, IL-21, we interrogate the expression of other pTOF panel markers (**Figure 6D**). Expression of stress and proliferation proteins pERK, IKB, pS6, pPLCg2, and CD24 is associated with T cells with high expression of pSTAT1 in IFN-H patients. Expression of CD45RA, CD27 and CD127 is associated with T cells with high expression of either pSTAT1 or pSTAT3 in IFN-H patients. Expression patterns of pTOF markers are not specific to IFN-H patients or stimulation condition (**Supplementary Figure 4**).

## Discussion

The recent FDA approval of anifrolumab for the treatment of SLE, represents an important advancement in the field. Despite this milestone, the mechanisms by which IFN signaling perturbs other cytokine pathways in the setting of a highly dysregulated SLE immune system, remains unclear. Here we apply highly multiplex single cell mass cytometry to elucidate the effects of the IFN signature on critical SLE-associated cytokines across multiple immune cell subsets. We report a robust IFN signature in SLE patients that is strongly associated with more severe disease, renal and CNS involvement and autoantibody production. Furthermore, our CyTOF analysis reveals increased STAT1/3/5 phosphorylation in IFN-H patients and phosphorylation of STAT proteins not typically utilized downstream of IFNα, IFNγ, and IL-21 signaling. Together our results, support a pathogenic role of IFN signature that is associated with increased phosphorylation of both common and uncommon STAT proteins.

Increased autoantibody production in IFN-H patients has been previously described (29), but differences in reactivity between IFN-H and IFN-L patients have been reported against histone-associated antigens. In contrast, autoantibody profiling of the Stanford SLE cohort displays significantly different levels of autoantibodies against spliceosome components between IFN-H and IFN-L patients. The U1-snRNP immune complex can stimulate macrophage migration inhibitory factor (MIF) production from monocytes and macrophages (43), and elevated MIF levels have been associated with more severe SLE disease (44). Because our mass cytometry panels skew towards lymphoid rather than myeloid lineages, we are unable to interrogate the relationship between autoantibody production against spliceosome complements and myeloid cell signaling.

The discrepancy between histone-associated and spliceosome-associated antigens across studies is likely cohort specific and possibly driven by the vastly different ethnic makeup of each cohort. Previous studies investigated cohorts with larger representation of African American patients as compared to higher representation of Asian (27%) and Latino (23%) patients in the Stanford SLE cohort. Genetic factors play a role in SLE development (45, 46), and we hypothesize that pathogenic epigenetic modifications and or allelic polymorphisms differ among ethnic groups. Our results do support a higher percentage of patients with the IFN signature in ethnic minority groups - Asian or Latino in the Stanford cohort (**Table 1**) and African American in previous studies.

Our phenotypic profiling of SLE patient PBMCs with single cell mass cytometry using our pTOF and ICTOF panels, demonstrate differences in the abundance of immune cell subsets. IFN-H patients have higher percentages of B cells and lower percentages of NK cells as compared to IFN-L patients. The increased percentage of B cells supports our finding that IFN-H patients display increased autoantibody production and an established finding of increased B cell subsets (including class-switched memory B cells and plasmablasts) in SLE PBMCs compared to HCs (47). Type I IFN can also activate and promote proliferation of multiple B cell subsets (48, 49). The frequency and role of NK cells in SLE is less well-studied, in large part due to NK cell diversity and divergent roles of peripheral versus organ- or tissue- trafficked NK cells. Studies of both murine models of SLE and SLE patients report decreased frequency of circulating NK cells in more severe disease (50, 51) and therefore a protective role in SLE. However, kidney-infiltrating NK cells have also been reported in SLE mice with more severe kidney disease (52), suggesting a deleterious role in SLE. Our study of PBMCs supports previous reports of lower NK cell numbers correlating with more severe disease.

Various T cell subsets have been implicated in playing a role in SLE pathogenesis. These include Tregs, Th17 and Tfhs. Our ICTOF panel analysis allows for identification of these subsets. While previous studies have shown both decreased (53, 54) or unchanged (55) frequencies of Tregs in SLE patients compared to HCs, here we report decreased abundance of Tregs in PBMCs of SLE patients as compared to HCs, but no notable difference between IFN-H and IFN-L patients (pTOF and ICTOF panels). Our analysis of peripheral Tfhs shows an increased abundance in IFN-L patients as compared to HCs, and a decreased abundance in IFN-H patients as compared to HCs. While the relationship between IFN signature and Tfh biology has not been thoroughly investigated, current dogma is that Tfhs are expanded in SLE as compared to HCs (56). While no major differences are seen in the abundance of Th17s across groups, it is worth noting that the frequencies of T cell subsets are exceedingly low in our study and our sampling represents patients with well controlled disease (patients receiving the equivalent of more than 10mg of prednisone daily or biologic therapies were excluded from CyTOF analysis).

Our MetaCyto and OMIQ analysis of phosphorylated intracellular signaling molecules (pTOF) demonstrates a significant effect of IFN signature on STAT phosphorylation. Phosphorylated STAT1, 3, and 5 are included in this study and all have been reported to play pathogenic roles in SLE (57). JAK/STAT inhibitor baricitnib (targeting STAT1/3) has shown clinical efficacy in the treatment of SLE (58) and others are currently being studied. As expected, our study reports increased phosphorylation of canonical STAT1 protein downstream of IFNα in IFN-H versus IFN-L patients. These results further establish the pathogenic role of IFN signaling in SLE.

Unexpectedly, IFN-H patients also show increased expression of pSTAT3 (Y705 – phosphorylation of tyrosine at position 705) and pSTAT5 (Y694 – phosphorylation of tyrosine at position 694) downstream of IFNα. As mentioned, IFNα-induced STAT3 and STAT5 phosphorylation in less frequent, and neither are involved in the formation of the ISGF3 complex (comprised of STAT1, STAT2, and IRF9) that is responsible for transactivation of IFN-mediated genes. Previous work has suggested distinct function of mechanism of activation for both STAT3 and STAT5 in IFNα signaling (59). STAT3 has been reported to negatively regulate type I IFN signaling by sequestering STAT1 and suppressing the formation of DNA-binding STAT1 homodimers (60) and *in vitro* STAT3 knockout leads to increased expression of a distinct subset of IFN-mediated genes (61). Additionally, STAT3 activation can be regulated by microRNAs like mir-155 (62, 63) and mir-221 (64). Abrogation of STAT1 leads to increased STAT3 phosphorylation in macrophages and in mouse models of SLE (37, 65), however the conditions leading to STAT3 phosphorylation with intact STAT1 remains unknown. IFNα-mediated STAT5 phosphorylation has also been reported *via* both serine and tyrosine phosphorylation, in addition to forming a complex with CrkL – an adaptor protein responsible for linking proteins in various signaling cascades (66).

While our study measures the primary mechanism of STAT activation through tyrosine phosphorylation, other mechanisms including acetylation, methylation and SUMOlyation are not represented. Furthermore, unphosphorylated STAT proteins are not included in our pTOF panel and future studies interrogating the ratio of unphosphorylated to phosphorylated STAT proteins along with inhibitory SOCs proteins could provide more insight into signaling dynamics. How and under what conditions less common STAT proteins are phosphorylated in SLE remains unknown and is ripe for investigation.

Our study confirms the critical role of IFN signaling and IFN signature in the pathogenesis of SLE. It also suggests that IFN signaling in SLE leads to increased and aberrant STAT phosphorylation. Of note, recent work utilizing CyTOF in mycophenolate mofetil (MMF)-treated SLE patients, suggests its efficacy is mediated through inhibition of pSTAT3 (67). Taken together with our current study, these results could support a combinatorial therapeutic approach targeting both IFN signature and STAT phosphorylation. Lastly, our study holds crucial implications for therapeutic approaches with JAK/STAT inhibitors currently under study in addition to elucidating the mechanisms by which STAT activation is regulated in SLE.

## Materials and Methods

### Patients

All SLE patients were diagnosed by the American College of Rheumatology SLE updated 1997 criteria (68). Samples were collected at the outpatient clinics at Department of Rheumatology, Stanford University Hospital. At the initial visit patients were screened for dsDNA, anti-Smith antibodies, and anti-nuclear antibodies (ANA). The SLEDAI was calculated at the time of sampling. EDTA stabilized serum samples from SLE patients and healthy controls were stored at -80°C until use. For interferon alpha profiling, RNA was collected from SLE patients (n=81) and healthy controls (HC) (n=31) in Tempus tubes (Applied Biosystems), processed and stored at -80 until use. For CyTOF studies, peripheral blood mononuclear cells (PBMCs) from selected SLE patients (n=31) and HC (n=17) were purified using Ficoll-Paque (GE Healthcare) and density centrifugation and frozen in RPMI-1640 with 20% FCS, and 10% DMSO at -150°C until use. Patients receiving any biologics (e.g. Belimumab or similar), prednisone (>10mg/day), MMF, or azathioprine were excluded from CyTOF analysis. Demographics of patients included in IFN profiling or CyTOF analysis are shown in **Table 1**. Demographics of HCs are unknown as HCs were anonymous.

### Interferon Alpha Profiling

Interferon alpha profiles were measured using a panel of IFN alpha target genes. These genes were selected from 5 previously validated IFN profile panels (5, 6, 29–31). Genes were selected based on appearances in 2 or more of these panels yielding a panel of 44 transcripts (**Supplementary Table 1**). The interferon score was calculated as previously described by Baechler *et al.* In brief, the individual transcripts were normalized to the highest values across all samples and a cumulative score of the normalized value for each transcript was calculated for all patients. Multiple alternative approaches to calculating this score were attempted and they all yield slightly different distributions but were very consistent in the ranking of the individual patients. For 10-50 ng total RNA of each sample, reverse transcription of the RNA to cDNA was performed at 50°C for 15 minutes using the High-Capacity Reverse Transcription kit (ABI). RT was performed directly on a 96-well PCR plate (ABI). PreAmp was performed on a thermocycler using the TaqMan PreAmp Master Mix Kit (Invitrogen) added to cDNA and pooled Taqman assays. RT enzyme was inactivated and the Taq polymerase reaction was started by bringing the sample to 95°C for 2 minutes. The cDNA was preamplified by denaturing for 10 cycles at 95°C for 15 seconds, annealing at 60°C for 4 minutes. The resulting cDNA product was diluted 1:2 with 1X TE buffer (Invitrogen). 2X Applied Biosystems Taqman Master Mix, Fluidigm Sample Loading Reagent, and preamplified cDNA were mixed and loaded into the 48.48 Dynamic Array (Fluidigm) sample inlets, followed by loading 10X assays into the assay inlets. Manufacturer’s instructions for chip priming, pipetting, mixing, and loading onto the BioMark system were followed. Real-time PCR was carried out with the following conditions: 10 min at 95°C, followed by 50 cycles of 15 sec at 95°C and 1 min at 60°C. Data was analyzed using Fluidigm software. All reactions were performed in duplicate, and Ct values were normalized to the geomean of GAPDH, ACTB, and B2M. Repeat positive controls were included across all chips.

### Lupus Autoantigen Microarrays

Detailed autoantibody profiling protocols and a list of arrayed antigens have been previously published (34). Briefly, 24 SLE-associated autoantigens and controls were printed at 0.2 mg/ml in ordered arrays on nitrocellulose-coated FAST slides (Whatman, Piscataway, New Jersey) using a VersArray ChipWriter Pro Robotic Arrayer (Bio-Rad). Individual arrays were blocked with PBS containing 3% FCS and 0.05% Tween 20 (Sigma-Aldrich) for 1.5 hrs on a rocking platform at room temperature. Arrays were probed with 400 μl human serum diluted 1:250 in 1X PBST with 5% FCS for 1.5 hrs on a rocking platform at 4°C, followed by washing and incubation with a 1:2000 dilution of cyanine-3-conjugated goat anti-human IgM or IgG secondary antibody (Jackson ImmunoResearch Laboratories). Arrays were scanned using a GenePix 4000B scanner (Molecular Devices) at constant PMT power for all arrays. The net mean pixel intensities of each feature were determined using GenePix Pro 6.1 software (Molecular Devices, Sunnyvale, California). Array data will be uploaded to the GEO database upon publication of the manuscript.

### Enzyme-Linked Immunosorbent Assays

96 well plates (NUNC MaxiSorp) were incubated at 4°C overnight with 100 µL/well protein solution at 2µg/ml (Ro/SSA, ssDNA, EBV, U1-A, U1-C, U170, Sm/RNP, smith, dsDNA(plasmid) and histones). Plates were then washed 3X with phosphate buffered saline with 0.05% Tween 20 (PBST) and blocked with 200µL/well PBST with 5% FCS for 2 hrs at room temperature. After washing, plates were incubated with 100 µL/well of sample at dilutions 1:400 or 1:800 at 4°C overnight, followed by washing and development using Europium labeled anti-human IgG and DELFIA Enhancement solution (PerkinElmer). IFN-H, n=20; IFN-L, n=20, HC, n=3. Detection limit was calculated as 2X the standard deviation of the blanks.

### Mass Cytometry Intracellular and Phospho-Specific Staining

Cytometry Time-of-Flight (CyTOF) analyses were performed at the Human Immune Monitoring Center at Stanford University. Detailed protocol is available electronically: iti.stanford.edu/himc/protocols.html. In brief, PBMCs were thawed and viable cells were counted by Vicell. Cells were added to a V-bottom microtiter plate at 1.5 million viable cells/well and washed once in fresh CyFACS buffer. Appropriate stimulations were added and cultured at 37°C (ICS stimulation: PMA (phorbol 12-myristate 13-acetate; 50 ng/ml), ionomycin (Sigma, 750 ng/ml), brefeldin-A (Sigma, 20 ug/ml for 4 hours; phospho-specific stimulation: IFNα, IFNγ, IL-21 for 20 minutes). The cells were stained for 60 min on ice with 50 uL of antibody-polymer conjugate surface marker cocktail. After staining the cells were washed and resuspended in 100 uL 2% PFA in PBS and placed at 4°C overnight. The next day, the cells are permeabilized in 100 uL eBiosciences permeabilization buffer and placed on ice for 45 min before staining with intracellular cocktail for 1 hour on ice. The cells were washed twice and resuspended in 100 uL iridium-containing DNA intercalator (1:2000 dilution in PBS; Fluidigm) and incubated at room temperature for 20 min then resuspended in MilliQ water and injected into the CyTOF (Fluidigm). Mass cytometry antibodies can be found in **Supplementary Table 2**.

### Mass Cytometry Data Analysis

#### OMIQ

Cytometry data files were normalized using the bead-bead Fluidigm normalization algorithm. Files were then manually gated in FlowJo for stability of time (191^+^/Time), cells with no beads (Ir193^+^/Ce140^-^), cleanup (double positive for DNA), and singlets (Ir193^+^). Total single cells or T cells (ICTOF: CD33^-^/CD56^-^/C20^-^/CD19^-^/CD3^+^, and for pTOF: CD14^-^/CD56^-^/C20^-^/CD19^-^/CD3^+^) were exported for analysis utilizing the OMIQ platform (www.omiq.ai). Data was Arcsinh transformed with a coefficient of 5 was used inside OMIQ platform. For lineage populations analysis, total single cells were downsampled between 30,000 (ICTOF) and 35,000 (pTOF) events, followed by unsupervised uniform manifold approximation and projection (UMAP) and FlowSOM algorithms. Results were plotted using OMIQ platform. For analysis of the T regulatory (Tregs: CD25^+^CD127^-^), T follicular (Tfh: CXCR5^+^PD1^+^), and Th17 (CD45RA^-^IL17^+^) cell subsets, manual gating of CD4 T cells individual samples was performed using OMIQ.

#### MetaCyto

We used the MetaCyto guided analysis pipeline (42) to evaluate cell populations using pre-defined marker definitions (**Supplementary Table 3**). Statistical analysis was performed using a fixed-effects multiple regression model (Marker ~ Group + Treatment + Study ID). Default parameters were used. Transformed marker values less than a 0.125 threshold were set to 0. Effect size was calculated by dividing the regression coefficient by the standard deviation of Marker. P values were adjusted using Benjamini-Hochberg false discovery rate (FDR).

### Statistics

Microarray data were expressed as mean net fluorescence intensity (MFI) units, representing the mean values from six replicate antigen features on each array. Non-reactive samples were defined as having a maximum normalized IgG MFI of less than 1,000 for a given antigen. Significance Analysis of Microarrays (SAM) (38) was applied to the dataset (with the MFI value of undetected array features set to 1) using the Wilcoxon signed-rank test statistic to identify antigens or cytokines/chemokines with statistically significant differences in array reactivity between different groups of mice at FDR of 0 (q<0.001). Binding reactivity heatmaps were generated using MultiExperiment Viewer (MEV TM4 Microarray Software Suite version 10.2, Dana-Farber Cancer Institute, Boston, MA) using k-nearest neighbor replacement and average linkage using Euclidean distance hierarchical clustering.

Statistical analyses for clinical data were performed using GraphPad Prism 9.2.0 for Mac (GraphPad Software). All data in text or graphs are expressed as medians with interquartile ranges unless otherwise specified. Non-paired, non-parametric data was analyzed by Mann-Whitney test. Correlation of non-parametric paired data was tested using Spearman’s Rho. In all tests the level of significance was a two-sided p value of less than 0.05.

## Data Availability Statement

The datasets presented in this study can be found in online repositories. The names of the repository/repositories and accession number(s) can be found below: GEO, GSE193174.

## Ethics Statement

The studies involving human participants were reviewed and approved by IRB# 17374: Stanford University Immunological and Rheumatic Disease Database: Disease Activity and Biomarker Study. The patients/participants provided their written informed consent to participate in this study.

## Author Contributions

Conceptualization, GY, TR, and PU. Methodology, GY and TR. Formal analysis, GY, TR, BT, BC-A, and GD. Investigation, GY, TR, BT, BC-A, JT, DH, VD, and GD. Data Curation, GY, TR, BT, BC-A, and JT. Writing – Original Draft, GY and PU. Writing – Review and Editing, GY, TR, BT, BC-A, BD, GC, and PU. Supervision, PU. Funding acquisition, P.J.U. All authors contributed to the article and approved the submitted version.

## Funding

PU was supported by NIH U19-AI110491 and R01 AI125197-01; the Donald E. and Delia B. Baxter Foundation; and the Henry Gustav Floren Trust. The contents are solely the responsibility of the authors and do not necessarily represent the official views of the NIH or one of its institutes. BT acknowledges the support of the University of California, Los Angeles, Caltech Medical Scientist Training Program. GY acknowledges the support of the Stanford Immunology Graduate Program and Stanford Medical Scientist Training Program.

## Conflict of Interest

The authors declare that the research was conducted in the absence of any commercial or financial relationships that could be construed as a potential conflict of interest.

## Publisher’s Note

All claims expressed in this article are solely those of the authors and do not necessarily represent those of their affiliated organizations, or those of the publisher, the editors and the reviewers. Any product that may be evaluated in this article, or claim that may be made by its manufacturer, is not guaranteed or endorsed by the publisher.

## Supplementary Material

Supplementary Table 1
**(related to Figure 1):** IFN profiling. IFN biosignature profiling. Fluidigm multiplex qPCR measured 44 transcripts. Transcripts were chosen based on a literature query for transcript profiling of bulk PBMCs or magnetically sorted immune subsets from SLE patients.

Supplementary Table 2
**(related to Figure 4–6):** Mass cytometry antibodies. Antibodies used for mass cytometry panels. Intracellular cytokine CyTOF panel (ICTOF) and phosphorylation of signaling protein CyTOF panel (pTOF). FLUIDIGM = FLUIDIGM Sciences, In house = antibody was conjugated and titrated in house. Grey box indicates use in panel.

Supplementary Table 3
**(related to Figure 5):** MetaCyto cell marker definitions. Marker definition of cell subsets for MetaCyto analysis – pTOF and ICTOF.

Supplementary Figure 1
**(related to Figure 1):** The IFN biosignature was calculated using our consensus 44 transcript panel showed significant correlation to the Baechler transcripts (rho=0.98, p<0.0001, left) and the Behrens (rho=0.92, p<0.0001, right) transcripts.

Supplementary Figure 2
**(related to Figure 4): (A–E)** Immune cell subsets identified by unsupervised OMIQ reveal differences in abundance between IFN-H and IFN-L patients. **(A)** Unsupervised UMAP pTOF analysis identifies multiple immune cell subsets (T cells, B cells, NK cells, NKT cells, myeloid cells, monocytes) across stimulation conditions (US = unstimulated, IFNα, IFNγ, and IL-21). **(B)** Unsupervised UMAP ICTOF analysis identifies multiple immune cell subsets (T cells, B cells, NK cells, γδ T cells, and myeloid cell) across stimulation conditions (US = unstimulated and PMA and ION = ionomycin. **(C)** Unsupervised UMAP ICTOF analysis of T cells, identifies multiple T cell subsets (CD4^+^ T cells, DP = double positive, and DN = double negative T cells) across conditions in **(C)**. **(D)** Abundance of immune cell subsets in **(B)** shows decreased T cells and increased B cells in IFN-H PBMCs stimulated with PMA+ION compared to HC and IFN-L PBMCs. **(E)** Abundance of T cell subsets in **(C)**. reveal decreased Treg and Tfh populations in IFN-H PBNCs stimulated with PMA+ION compared to HC and IFN-L PBMCs. All T regulatory (Tregs: CD25^+^CD127^-^), T follicular (Tfh: CXCR5^+^PD1^+^), and Th17 (CD45RA^-^IL17^+^) populations above were hand-gated within OMIQ. Treg, Tfh and Th17 populations were omitted from T cell UMAP in **C** due to rarity of populations.

Supplementary Figure 3
**(related to Figure 5): (A–D)** MetaCyto analysis of pTOF and ICTOF panels. **(A)** Dotmap represents effect size of IFN signature (IFN-H v. IFN-L patients) or **(B)** SLE, on signaling protein (columns) across cell subsets (rows) after stimulation with IFNα (left), IFNγ (middle), or IL-21 (right). **(C)** Dotmap represents effect size of IFN signature (IFN-H v. IFN-L patients, right) or **(B)** SLE (left), on intraceullar protein production (columns) across cell subsets (rows) after stimulation with PMA and ionomycin. Dot size depicts effect size, larger dots have greater effect sizes. Color depicts direction of effect size, red indicates positive effect size and blue indicates negative effect size. Shading of each box depicts statistical significance by -log10(FDR), darker boxes indicate greater statistical significance. Only cell populations with FDR < 0.05 are displayed in the dotmaps. **(D)** Forest plots representing the effect size of IFN signature (IFN-H v. IFN-L) on phosphylation of pSTAT5 downstream of IFNα (left), IFNγ (middle), or IL-21 (right) stimulation. Significant (p<0.05) effect sizes in red and not significant effect sizes in black. P values were adjusted using Benjamini-Hochberg false discovery rate (FDR).

Supplementary Figure 4
**(related to Figure 6):** Histograms displaying expression of both surface (CD19, CD24, CD27, and CD127) and intracellular signaling (pSTAT1, pSTAT3, pSTAT5, pERK, IKB, pS6, and pPLCg2) proteins of manually gated T cells expressing pSTAT1 and 3, pSTAT1, pSTAT3, or DN = double negative T cells, from HC, IFN-L or IFN-H PBMCs stimulated with IFNα, IFNγ, IL-21 or unstimulated (US).

## Abbreviations

IFN, interferon; SLE, Systemic Lupus Erythematosus; HC, healthy control; PBMC, peripheral blood mononuclear cell; IFN-H, interferon-high; IFN-L, interferon-low; IFNAR, subunit 1 of the type I interferon receptor (IFNAR1); BICLA, British Isles Lupus Assessment Group-based Composite Lupus Assessment; STAT, signal transduction and activators of transcription; JAK, janus family tyrosine kinase; LN, lupus nephritis; ANA, anti-nuclear antibody; ELISA, enzyme-linked immunosorbent assay; PMA, phorbol 12-myristate 13-acetate; ION, ionomycin; CyTOF, cytometry by time of flight; pTOF, phosphorylation of intracellular signaling proteins TOF; ICTOF, intracellular cytokine TOF; Th17, T helper 17; Tfh, T follicular helper; Treg, regulatory T; NK, natural killer; SAM, significance analysis of microarrays; FDR, false discovery rate; MFI, mean fluorescence intensity; UMAP, unsupervised uniform manifold approximation and projection; US, unstimulated.
