# Supplementary material for: High Interferon Signature Leads to Increased STAT1/3/5 Phosphorylation in PBMCs From SLE Patients by Single Cell Mass Cytometry
Source: Front Immunol. 2022 Jan 28;13:833636. doi: 10.3389/fimmu.2022.833636 (PMC8851522; doi:10.3389/fimmu.2022.833636)
Supplement: Supplementary Figure 1 — (related to Figure 1): The IFN biosignature was calculated using our consensus 44 transcript panel showed significant correlation to the Baechler transcripts (rho=0.98, p<0.0001, left) and the Behrens (rho=0.92, p<0.0001, right) transcripts. [file Image_1.pdf]

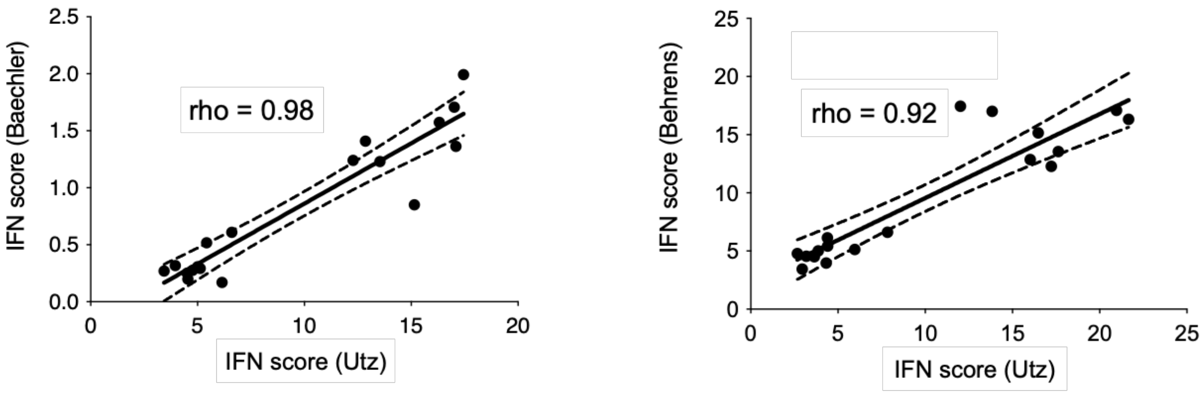

Supplementary Figure 1 (related to Figure 1): The IFN biosignature was calculated using our consensus 44 transcript panel showed significant correlation to the Baechler transcripts (rho=0.98, p<0.0001, left) and the Behrens (rho=0.92, p<0.0001, right) transcripts.
