# Supplementary material for: High Interferon Signature Leads to Increased STAT1/3/5 Phosphorylation in PBMCs From SLE Patients by Single Cell Mass Cytometry
Source: Front Immunol. 2022 Jan 28;13:833636. doi: 10.3389/fimmu.2022.833636 (PMC8851522; doi:10.3389/fimmu.2022.833636)
Supplement: Supplementary Figure 2 — (related to Figure 4): (A–E) Immune cell subsets identified by unsupervised OMIQ reveal differences in abundance between IFN-H and IFN-L patients. (A) Unsupervised UMAP pTOF analysis identifies multiple immune cell subsets (T cells, B cells, NK cells, NKT cells, myeloid cells, monocytes) across stimulation conditions (US = unstimulated, IFNα, IFNγ, and IL-21). (B) Unsupervised UMAP ICTOF analysis identifies multiple immune cell subsets (T cells, B cells, NK cells, γδ T cells, and myeloid cell) across stimulation conditions (US = unstimulated and PMA and ION = ionomycin. (C) Unsupervised UMAP ICTOF analysis of T cells, identifies multiple T cell subsets (CD4+ T cells, DP = double positive, and DN = double negative T cells) across conditions in (C). (D) Abundance of immune cell subsets in (B) shows decreased T cells and increased B cells in IFN-H PBMCs stimulated with PMA+ION compared to HC and IFN-L PBMCs. (E) Abundance of T cell subsets in (C). reveal decreased Treg and Tfh populations in IFN-H PBNCs stimulated with PMA+ION compared to HC and IFN-L PBMCs. All T regulatory (Tregs: CD25+CD127-), T follicular (Tfh: CXCR5+PD1+), and Th17 (CD45RA-IL17+) populations above were hand-gated within OMIQ. Treg, Tfh and Th17 populations were omitted from T cell UMAP in C due to rarity of populations. [file Image_2.pdf]

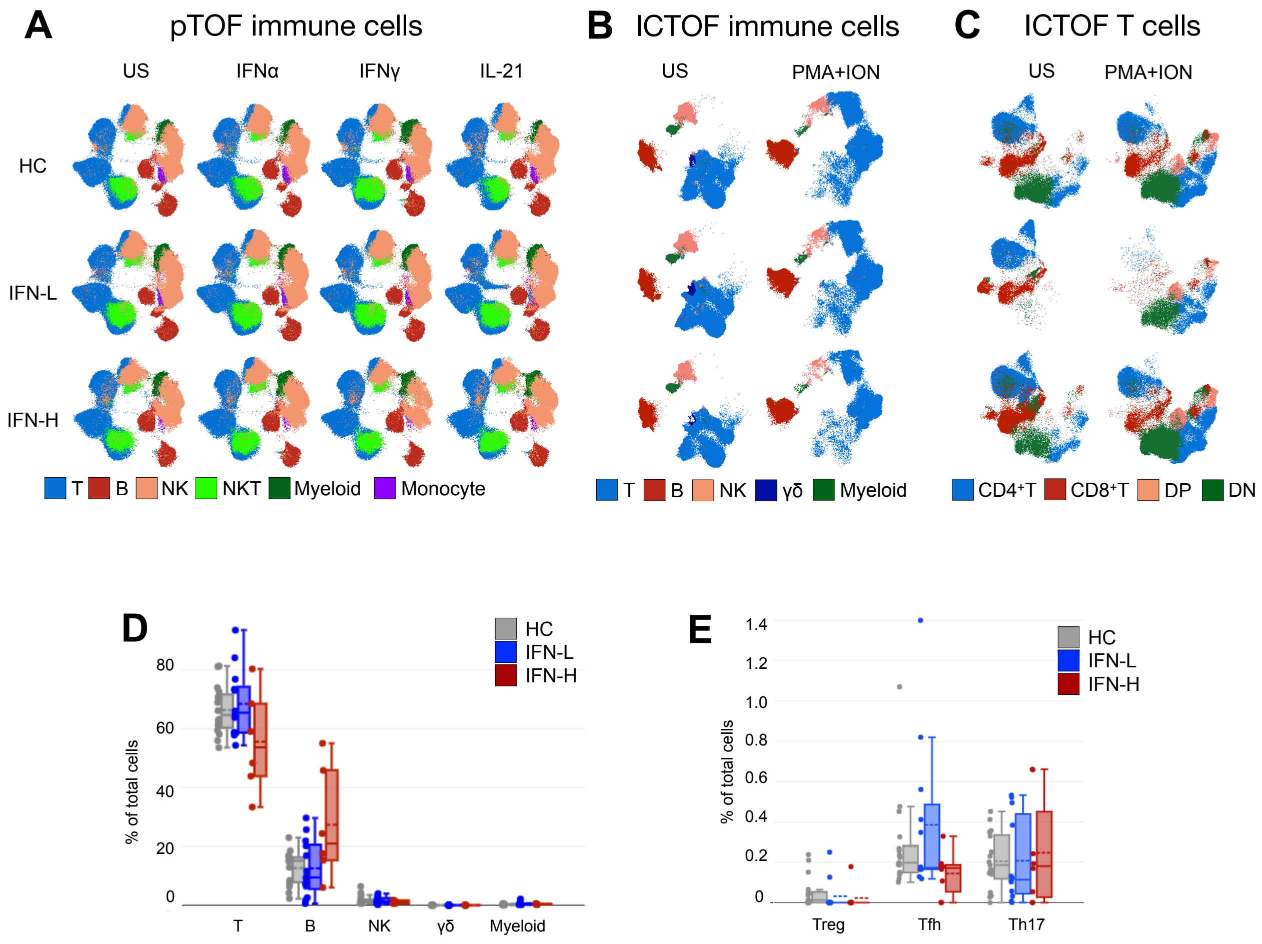

Supplementary Figure 2 (related to Figure 4): (A-E) Immune cell subsets identified by unsupervised OMIQ reveal differences in abundance between IFN-H and IFN-L patients. **(A)** Unsupervised UMAP pTOF analysis identifies multiple immune cell subsets (T cells, B cells, NK cells, NKT cells, myeloid cells, monocytes) across stimulation conditions (US = unstimulated, IFNα, IFNγ, and IL-21). **(B)** Unsupervised UMAP ICTOF analysis identifies multiple immune cell subsets (T cells, B cells, NK cells, γδ T cells, and myeloid cell) across stimulation conditions (US = unstimulated and PMA and ION = ionomycin. **(C)** Unsupervised UMAP ICTOF analysis of T cells, identifies multiple T cell subsets ($CD4^+$ T cells, DP = double positive, and DN = double negative T cells) across conditions in **B**. **(D)** Abundance of immune cell subsets in **B** shows decreased T cells and increased B cells in IFN-H PBMCs stimulated with PMA+ION compared to HC and IFN-L PBMCs. **(E)** Abundance of T cell subsets in **C**. reveal decreased Treg and Tfh populations in IFN-H PBNCs stimulated with PMA+ION compared to HC and IFN-L PBMCs. All T regulatory (Tregs: $CD25^+CD127^-$), T follicular (Tfh: $CXCR5^+PD1^+$), and Th17 ($CD45RA^-IL17^+$) populations above were hand-gated within OMIQ. Treg, Tfh and Th17 populations were omitted from T cell UMAP in **C** due to rarity of populations.
