# Supplementary figures and images for: High Interferon Signature Leads to Increased STAT1/3/5 Phosphorylation in PBMCs From SLE Patients by Single Cell Mass Cytometry
Source: Front Immunol. 2022 Jan 28;13:833636. doi: 10.3389/fimmu.2022.833636 (PMC8851522; doi:10.3389/fimmu.2022.833636)

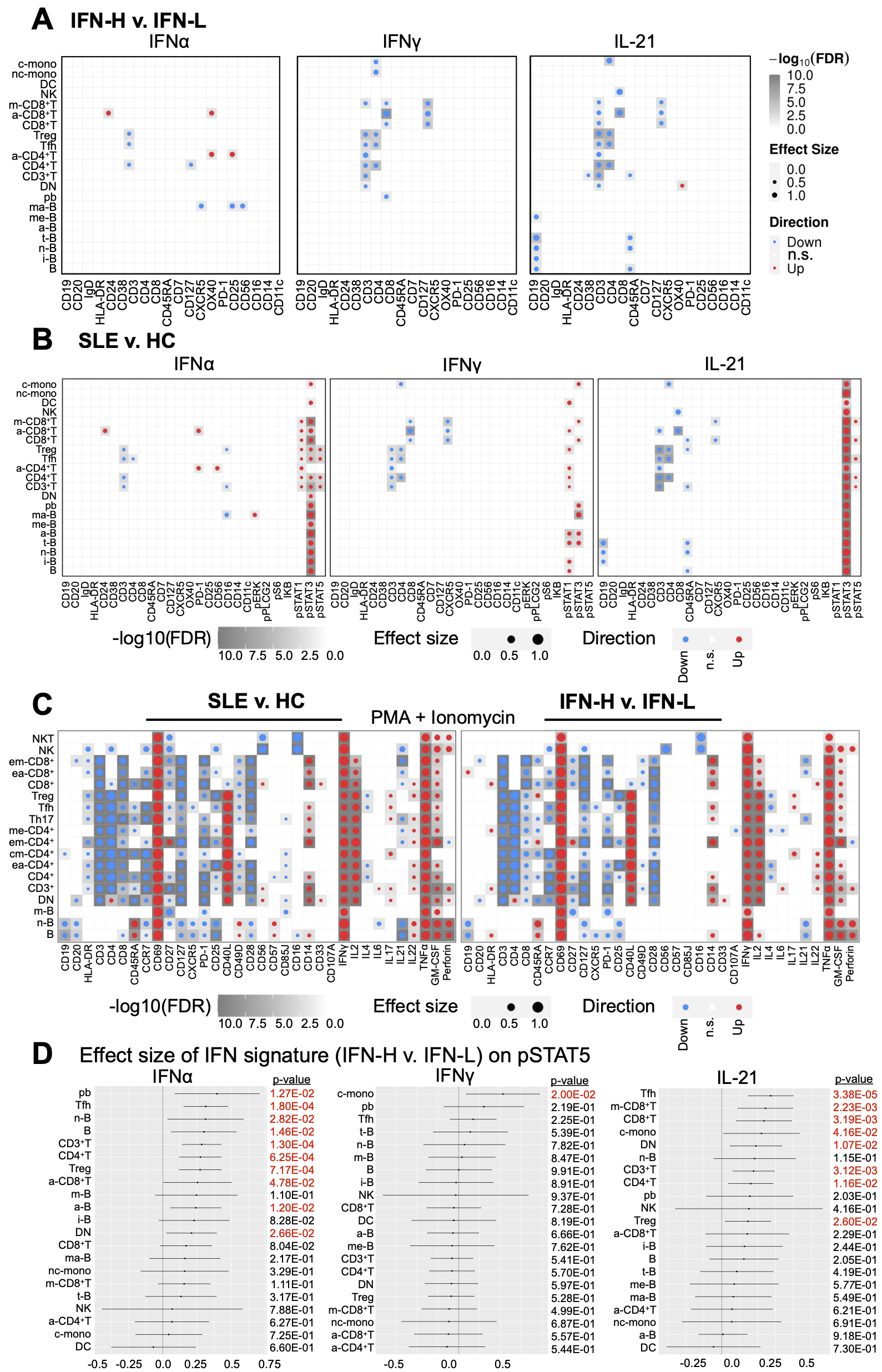

Supplement: Supplementary Figure 3 — (related to Figure 5): (A–D) MetaCyto analysis of pTOF and ICTOF panels. (A) Dotmap represents effect size of IFN signature (IFN-H v. IFN-L patients) or (B) SLE, on signaling protein (columns) across cell subsets (rows) after stimulation with IFNα (left), IFNγ (middle), or IL-21 (right). (C) Dotmap represents effect size of IFN signature (IFN-H v. IFN-L patients, right) or (B) SLE (left), on intraceullar protein production (columns) across cell subsets (rows) after stimulation with PMA and ionomycin. Dot size depicts effect size, larger dots have greater effect sizes. Color depicts direction of effect size, red indicates positive effect size and blue indicates negative effect size. Shading of each box depicts statistical significance by -log10(FDR), darker boxes indicate greater statistical significance. Only cell populations with FDR < 0.05 are displayed in the dotmaps. (D) Forest plots representing the effect size of IFN signature (IFN-H v. IFN-L) on phosphylation of pSTAT5 downstream of IFNα (left), IFNγ (middle), or IL-21 (right) stimulation. Significant (p<0.05) effect sizes in red and not significant effect sizes in black. P values were adjusted using Benjamini-Hochberg false discovery rate (FDR). [file Image_3.tiff]
