# Supplementary material for: High Interferon Signature Leads to Increased STAT1/3/5 Phosphorylation in PBMCs From SLE Patients by Single Cell Mass Cytometry
Source: Front Immunol. 2022 Jan 28;13:833636. doi: 10.3389/fimmu.2022.833636 (PMC8851522; doi:10.3389/fimmu.2022.833636)
Supplement: Supplementary Figure 4 — (related to Figure 6): Histograms displaying expression of both surface (CD19, CD24, CD27, and CD127) and intracellular signaling (pSTAT1, pSTAT3, pSTAT5, pERK, IKB, pS6, and pPLCg2) proteins of manually gated T cells expressing pSTAT1 and 3, pSTAT1, pSTAT3, or DN = double negative T cells, from HC, IFN-L or IFN-H PBMCs stimulated with IFNα, IFNγ, IL-21 or unstimulated (US). [file Image_4.pdf]

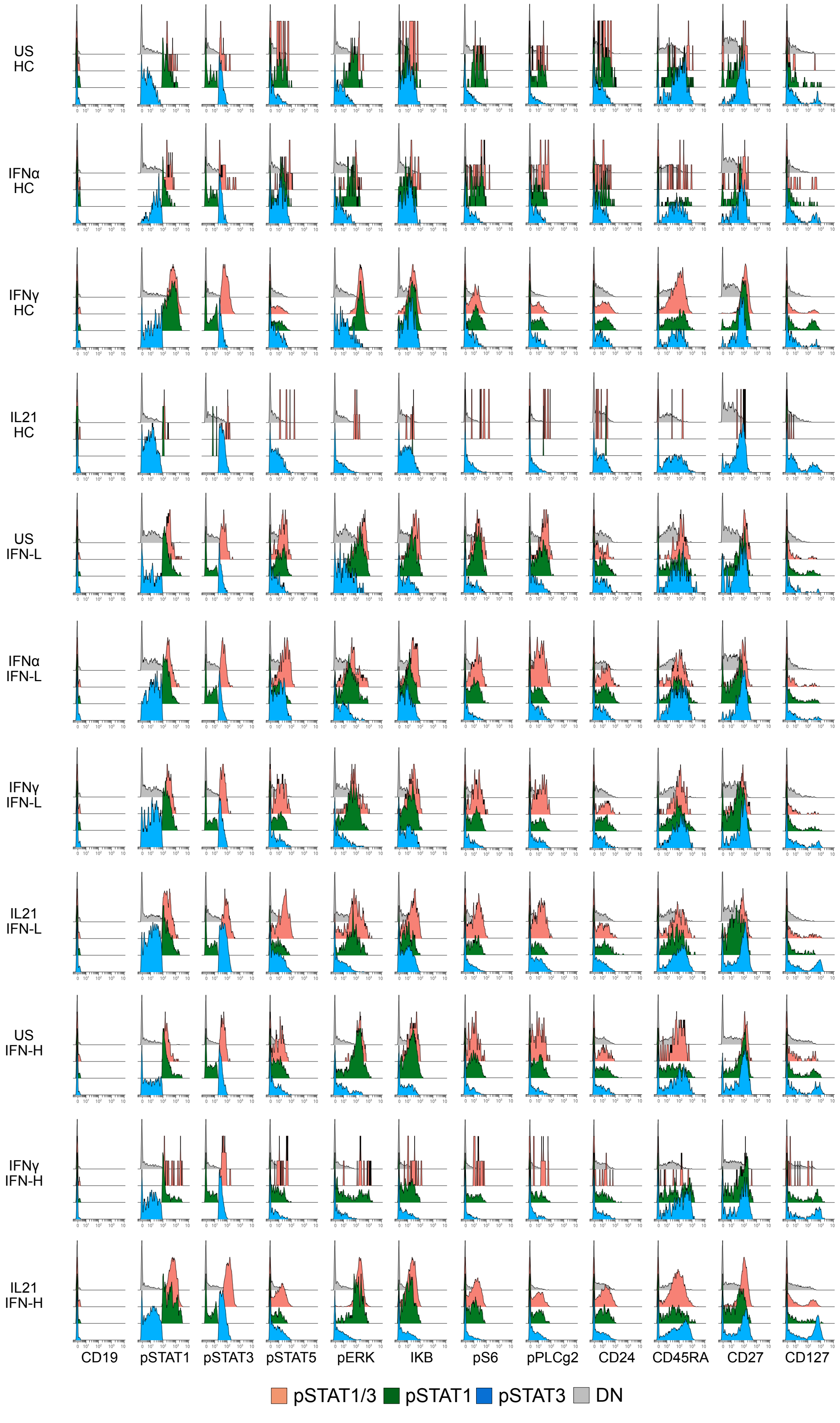
US
HC
IFNα
HC
IFNγ
HC
IL21
HC
US
IFN-L
IFNα
IFN-L
IFNγ
IFN-L
IL21
IFN-L
US
IFN-H
IFNγ
IFN-H
IL21
IFN-H
CD19
pSTAT1
pSTAT3
pSTAT5
pERK
IKB
pS6
pPLCg2
CD24
CD45RA
CD27
CD127
pSTAT1/3
pSTAT1
pSTAT3
DN

**Supplementary Figure 4 (related to Figure 6):** Histograms displaying expression of both surface (CD19, CD24, CD27, and CD127) and intracellular signaling (pSTAT1, pSTAT3, pSTAT5, pERK, IKB, pS6, and pPLCg2) proteins of manually gated T cells expressing pSTAT1 and 3, pSTAT1, pSTAT3, or DN = double negative T cells, from HC, IFN-L or IFN-H PBMCs stimulated with IFNα, IFNγ, IL-21 or unstimulated.
